# Supplementary material for: Infection and vaccination status of COVID-19 among healthcare professionals in academic platform: Prevision vs. reality of Bangladesh context
Source: PLoS One. 2022 Feb 18;17(2):e0263078. doi: 10.1371/journal.pone.0263078 (PMC8856526; doi:10.1371/journal.pone.0263078)
Supplement: S1 File — (DOCX) [file pone.0263078.s001.docx]

**Questionnaire link:**

<https://docs.google.com/forms/d/1aEeT9krJHRtCYas2u-1L15AyO5QnD_St0cGOSZqKyms/edit>

Top of Form

Urgent Info needed: Dept. of Public Health, NUB

NUB-MPH Survey for the COVID-19 infection status and campus choosing for classes

nasrin.ddc@gmail.com (not shared) [Switch account](mhtml:file://C:\Users\User\OneDrive\Desktop\Urgent%20Info%20needed_%20Dept.%20of%20Public%20Health,%20NUB.mhtml!https://accounts.google.com/AccountChooser?continue=https://docs.google.com/forms/d/1aEeT9krJHRtCYas2u-1L15AyO5QnD_St0cGOSZqKyms/viewform?edit_requested%3Dtrue&service=wise)

* Required

1. What is your name? *

Your answer

2. NUB ID:

Your answer

3. NUB batch number:

38 Batch

39 Batch

40 Batch

41 Batch

42 Batch

43 Batch

44 Batch

45 Batch

46 Batch

Clear selection

4. Phone number: *

Your answer

5. Please provide your e-mail ID:

Your answer

6. Gender: *

Female

Male

7. Age (In Years): *

Your answer

8. What is the name of your current working organization? *

Your answer

9. What is the type of your current working Organization? *

Public

Private

10. In which district is your current working Organization? *

Your answer

11. What is your current designation? *

Your answer

12. Working experience (In Years): *

Your answer

13. Have you been exposed with COVID-19 infection? *

Yes

No

14. Are you vaccinated (COVID-19)? *

Yes

No

15. If you are not vaccinated please write the reasons... *

Your answer

16. Where are you willing to attend the classes when the University will open?

City Campus at Kawranbazar (Address: Kawranbazar beside Farmgate , Dhaka)

Permanent Campus at Ashkona (Address: Ashkona (Near Haji Camp), Dhaka)

Clear selection

Submit

Clear form

Never submit passwords through Google Forms.

Bottom of Form

This content is neither created nor endorsed by Google. [Report Abuse](mhtml:file://C:\Users\User\OneDrive\Desktop\Urgent%20Info%20needed_%20Dept.%20of%20Public%20Health,%20NUB.mhtml!https://docs.google.com/forms/u/0/d/e/1FAIpQLSe7II8ab3VnCwNdOi_DkNJnw7J3LLMFW2xdLy4vPkECsKtTLg/reportabuse?source=https://docs.google.com/forms/d/e/1FAIpQLSe7II8ab3VnCwNdOi_DkNJnw7J3LLMFW2xdLy4vPkECsKtTLg/viewform?edit_requested%3Dtrue) - [Terms of Service](mhtml:file://C:\Users\User\OneDrive\Desktop\Urgent%20Info%20needed_%20Dept.%20of%20Public%20Health,%20NUB.mhtml!https://policies.google.com/terms) - [Privacy Policy](mhtml:file://C:\Users\User\OneDrive\Desktop\Urgent%20Info%20needed_%20Dept.%20of%20Public%20Health,%20NUB.mhtml!https://policies.google.com/privacy)
