# Supplementary material for: Infection and vaccination status of COVID-19 among healthcare professionals in academic platform: Prevision vs. reality of Bangladesh context
Source: PLoS One. 2022 Feb 18;17(2):e0263078. doi: 10.1371/journal.pone.0263078 (PMC8856526; doi:10.1371/journal.pone.0263078)
Supplement: Attachment — Submitted filename: Reviewer Comment_Plos One.docx [file pone.0263078.s004.docx]

**Reviewer’s Comments**

This study is interesting and important study for Bangladesh in fighting against COVID-19 outbreak. But, the manuscript still needs to improve the data analysis, presentation of the findings and implication of the study.

Background

- Need to describe the justification of the study clearly
- Study design with retrospective and structure: What does it mean?
- What is your study setting? Which responsibility did your participants take in which region? Are they still students or already graduated?
- In data analysis, need to describe how did you consider your final multivariable logistic model for each outcome variable
- When I check in the table 2, there are only 1 or 2 variables for AOR? Is it final model? Please explain.

Result session:

- In my opinion, 41% is not nearly half.
- Fig 1 and 2 should be pie chart. Figure 1 and 2 can be only one figure saying Fig 1.1 and 1.2. Fig 3 can also be combined to figure 2. Vaccination status and reasons for not having vaccines
- In my opinion, Table 1 and 2 should not be supplementary tables. Should describe as main table.
- Description of the results should be in text. Should not under the figure as picture.
- Regarding the result of preditors, I would like to suggest to describe the results from multivariable logistic regression with AOR.
- Did you add vaccine status as one of the predictor for COVID-19 infection?

Discussion

- Please discuss about 41% has infected. Please compare with other studies and what is the implication of your study? How will you apply?
- Please discuss about 81.7% has infected. Please compare with other studies and what is the implication of your study? How will you apply?
- Please describe the strength and limitation of your study.
- Please clearly describe the implication from your study.
- Please mention how you can make generalizability from your study population and sample size. Can we conclude this study represents all the health professional?
