# Supplementary material for: Infection and vaccination status of COVID-19 among healthcare professionals in academic platform: Prevision vs. reality of Bangladesh context
Source: PLoS One. 2022 Feb 18;17(2):e0263078. doi: 10.1371/journal.pone.0263078 (PMC8856526; doi:10.1371/journal.pone.0263078)
Supplement: Attachment — Submitted filename: Table file_Reviewer.docx [file pone.0263078.s005.docx]

| **Table 1: Characteristics of the respondents according to COVID-19 infection and vaccination status (n=300)** | | | | | | |  |  |
| --- | --- | --- | --- | --- | --- | --- | --- | --- |
| **Characteristics** | **COVID-19 infection status** | | | | **COVID-19 vaccination status** | | | |
|  | **Number of participants, n (%)** | **Test Positive, n (%)** | **Test negative, n (%)** | **p-value** | **Number of participants, n (%)** | **Vaccinated, n (%)** | **Non-vaccinated, n (%)** | **p-value** |
| **Age group (in years)** |  |  |  |  |  |  |  |  |
| ≤29 | 69 (23.0) | 19 (6.3) | 50 (16.7) | 0.01* | 69 (23.0) | 37 (12.3) | 32 (10.7) | 0.01* |
| 30-39 | 78 (26.0) | 33 (11.0) | 45 (15.0) |  | 78 (26.0) | 64 (21.3) | 14 (4.7) |  |
| 40-49 | 118 (39.3) | 49 (16.3) | 69 (23.0) |  | 118 (39.3) | 114 (38.0) | 4 (1.3) |  |
| >50 | 35 (11.7) | 22 (7.3) | 13 (4.3) |  | 35 (11.7) | 30 (10.0) | 5 (1.7) |  |
| **Gender** |  |  |  |  |  |  |  |  |
| Male | 67 (22.3) | 26 (8.7) | 41 (13.7) | 0.77 | 67 (22.3) | 47 (15.7) | 20 (6.7) | 0.01* |
| Female | 233 (77.7) | 97 (32.3) | 136 (45.3) |  | 233 (77.7) | 198 (66.0) | 35 (11.7) |  |
| **Profession** |  |  |  |  |  |  |  |  |
| Physicians | 44 (14.7) | 14 (4.7) | 30 (10) | 0.45 | 44 (14.7) | 32 (10.7) | 12 (4.0) | 0.01* |
| Nurses | 208 (69.3) | 87 (29.0) | 121 (40.3) |  | 208 (69.3) | 183 (61.0) | 25 (8.3) |  |
| Allied health professionals | 48 (16.0) | 22 (7.3) | 26 (8.7) |  | 48 (16.0) | 30 (10.0) | 18 (6.0) |  |
| **Experience** |  |  |  |  |  |  |  |  |
| ≤10 | 146 (48.7) | 51 (17.0) | 95 (31.7) | 0.04* | 146 (48.7) | 106 (35.3) | 40 (13.3) | 0.01* |
| >10 | 154 (51.3) | 72 (24.0) | 82 (27.3) |  | 154 (51.3) | 139 (46.3) | 15 (5.0) |  |
| **Type of Organization** |  |  |  |  |  |  |  |  |
| Private | 171 (57.0) | 74 (24.7) | 97 (32.3) | 0.45 | 171 (57.0) | 148 (49.3) | 23 (7.7) | 0.01* |
| Public | 129 (43.0) | 49 (16.3) | 80 (26.7) |  | 129 (43.0) | 97 (32.3) | 32 (10.7) |  |
| **Geographic Location** |  |  |  |  |  |  |  |  |
| Dhaka | 197 (65.7) | 84 (28.0) | 113 (37.7) | 0.57 | 197 (65.7) | 163 (54.3) | 34 (11.3) | 0.01* |
| Barisal | 24 (8.0) | 11 (3.7) | 13 (4.3) |  | 24 (8.0) | 23 (7.7) | 1 (0.3) |  |
| Chittagong | 46 (15.3) | 17 (5.7) | 29 (9.7) |  | 46 (15.3) | 29 (9.7) | 17 (5.7) |  |
| Khulna | 15 (5.0) | 3 (1.0) | 12 (4.0) |  | 15 (5.0) | 15 (5.0) | 0 (0.0) |  |
| Raj Shahi | 8 (2.7) | 3 (1.0) | 5 (1.7) |  | 8 (2.7) | 8 (2.7) | 0 (0.0) |  |
| Mymensingh | 10 (3.3) | 5 (1.7) | 5 (1.7) |  | 10 (3.3) | 7 (2.3) | 3 (1.0) |  |
| Data are presented as frequency (n), percentage (%); *Statistical significance at p value ≤0.05. Chi-square test was used to observe the association. | | | | | | | | |
|  |  |  |  |  |  |  |  |  |

| **Table 2. Identified predictors associated with the COVID-19 infection and vaccination status (n=300)** | | | | | | | | |
| --- | --- | --- | --- | --- | --- | --- | --- | --- |
| **Characteristics** | **COVID-19 infection status** | | | | **COVID-19 vaccination status** | | | |
|  | Test negative vs test positive counter | | | | Non-Vaccinated vs vaccinated counter | | | |
|  | Un-adjusted OR (95% CI) | P-value | Adjusted OR (95% CI) | P-value | Un-adjusted OR (95% CI) | P-value | Adjusted OR (95% CI) | P-value |
| **Age group (in years)** |  | | | |  | | | |
| ≤29 | 0.23 (0.09- 0.53) | 0.01* | 0.22 (0.09- 0.53) | 0.01* | 5.19 (1.80- 14.95) | 0.01* | 4.45 (1.51- 13.08) | 0.01* |
| 30-39 | 0.43 (0.19- 0.98) | 0.04* | 0.43 (0.19- 0.98) | 0.04* | 1.31 (0.43- 3.98) | 0.63 | 1.09 (0.35- 3.39) | 0.88 |
| 40-49 | 0.42 (0.19- 0.91) | 0.03* | 0.42 (0.19- 0.91) | 0.03* | 0.21 (0.05- 0.83) | 0.03* | 0.22 (0.06- 0.89) | 0.03* |
| >50 | Reference | | | | Reference | | | |
| **Gender** |  | | | |  | | | |
| Male | 0.89 (0.51- 1.55) | 0.68 | ─ | ─ | 2.41 (1.28- 4.54) | 0.01* | ─ | ─ |
| Female | Reference | | | | Reference | | | |
| **Profession** |  | | | |  | | | |
| Physicians | 0.55 (0.24- 1.29) | 0.17 | ─ | ─ | 0.63 (0.26-1.51) | 0.29 | 0.56 (0.21- 1.47) | 0.24 |
| Nurses | 0.85 (0.46- 1.59) | 0.61 | ─ | ─ | 0.23 (0.11-0.47) | 0.01* | 0.34 (0.15- 0.76) | 0.01* |
| Allied health professionals | Reference | | | | Reference | | | |
| **Experience** |  | | | |  | | | |
| ≤10 | 0.61 (0.38- 0.97) | 0.04* | ─ | ─ | 3.49 (1.84- 6.67) | 0.01* | ─ | ─ |
| >10 | Reference | | | | Reference | | | |
| **Type of Organization** |  | | | |  | | | |
| Private | 1.25 (0.78- 1.99) | 0.36 | ─ | ─ | 0.47 (0.26- 0.85) | 0.01* | ─ | ─ |
| Public | Reference | | | | Reference | | | |
| **Geographic Location** |  | | | |  | | | |
| Dhaka | 0.74 (0.21- 2.65) | 0.65 | ─ | 0.03* | 0.49 (0.12- 1.98) | 0.31 | ─ | ─ |
| Barisal | 0.85 (0.19- 3.71) | 0.83 | ─ | 0.08 | 0.10 (0.01- 1.14) | 0.06 | ─ | ─ |
| Chittagong | 0.59 (0.15- 2.32) | 0.45 | ─ | 0.04* | 1.36 (0.31- 6.01) | 0.68 | ─ | ─ |
| Khulna | 0.13 (0.04- 1.47) | 0.13 | ─ | ─ | ─ | ─ | ─ | ─ |
| Raj Shahi | 0.59 (0.09- 3.98) | 0.59 | ─ | ─ | ─ | ─ | ─ | ─ |
| Mymensingh | Reference | | | | Reference | | | |
| Logistic Regression Analysis was used to identify the predictors; * Statistical significance at p value ≤0.05. | | | | | | | | |
|  |  |  |  |  |  |  |  |  |
