# Supplementary material for: Infection and vaccination status of COVID-19 among healthcare professionals in academic platform: Prevision vs. reality of Bangladesh context
Source: PLoS One. 2022 Feb 18;17(2):e0263078. doi: 10.1371/journal.pone.0263078 (PMC8856526; doi:10.1371/journal.pone.0263078)
Supplement: Attachment — Submitted filename: Response to reviewer.docx [file pone.0263078.s006.docx]

| **Reviewer's Comment** | **Response** |
| --- | --- |
| **Background** | |
| 1. Need to describe the justification of the study clearly | Modified in line 71-86 |
| 2. Study design with retrospective and structure: What does it mean? | Modified in line 91-92 |
| 3. What is your study setting? Which responsibility did your participants take in which region? Are they still students or already graduated? | Already Mentioned in line 95-96 and 100-101 |
| 4. In data analysis, need to describe how did you consider your final multivariable logistic model for each outcome variable | Modified in line 133-134 |
| 5. When I check in the table 2, there are only 1 or 2 variables for AOR? Is its final model? Please explain. | Yes, it is the final model. We did the final model by backward elimination of the study variables. |
| **Result** | |
| 1. In my opinion, 41% is not nearly half. | Modified in line 149-50 |
| 2. Fig 1 and 2 should be pie chart. Figure 1 and 2 can be only one figure saying Fig 1.1 and 1.2. Fig 3 can also be combined to figure 2. Vaccination status and reasons for not having vaccines. | Modified as fig 1 and fig 2. We have merged fig 2 & 3 into new fig 2. |
| 3. In my opinion, Table 1 and 2 should not be supplementary tables. Should describe as main table. | Added in the result section |
| 4. Description of the results should be in text. Should not under the figure as picture. | Modified the figures |
| 5. Regarding the result of predators, I would like to suggest to describe the results from multivariable logistic regression with AOR. | Already mentioned in result section in line 180-186 |
| 6. Did you add vaccine status as one of the predictors for COVID-19 infection? | No, we did not find any association between vaccination status and infection status. |
| **Discussion** | |
| 1. Please discuss about 41% has infected. Please compare with other studies and what is the implication of your study? How will you apply? | Modified in lines 197-206 |
| 2. Please discuss about 81.7% has infected. Please compare with other studies and what is the implication of your study? How will you apply? | Modified in lines 207-219 |
| 3. Please describe the strength and limitation of your study. | Added in lines 257-265 |
| 4. Please clearly describe the implication from your study. |  |
| 5. Please mention how you can make generalizability from your study population and sample size. Can we conclude this study represents all the health professional? |  |
| **Editor's Comment** |  |
| Authors need to modify the data analysis, interpretation of results and to strengthen the discussion section. | Addressed in the track changed manuscript file |
| There are grammatical errors throughout and English language editing is deemed necessary. | Corrected and modified |
