# Supplementary material for: Infection and vaccination status of COVID-19 among healthcare professionals in academic platform: Prevision vs. reality of Bangladesh context
Source: PLoS One. 2022 Feb 18;17(2):e0263078. doi: 10.1371/journal.pone.0263078 (PMC8856526; doi:10.1371/journal.pone.0263078)
Supplement: Attachment — Submitted filename: Reviewer Comments_R1.docx [file pone.0263078.s007.docx]

**Reviewer’s comments_R1**

- What does it mean “retrospective approach” in line 83-84.

This cross-sectional study was carried out based on retrospective approach and the structured data were collected

- Need to mention more detail about the student participants to get clear presentation for readers. Not enough described in line 95-96 and 100-101.
- What is the total number of students in in summer-2021 semester (May to August 2021) of the MPH program of NUB? Are public health students are part-time students? Are they also working?
- The sample size said 384 but the study enrolled 300 students? What is your explanation for that?
- In data analysis, AOR should be used in stead of OR in multivariable logistic regression.
