# Supplementary material for: Infection and vaccination status of COVID-19 among healthcare professionals in academic platform: Prevision vs. reality of Bangladesh context
Source: PLoS One. 2022 Feb 18;17(2):e0263078. doi: 10.1371/journal.pone.0263078 (PMC8856526; doi:10.1371/journal.pone.0263078)
Supplement: Attachment — Submitted filename: Response to reviewer.docx [file pone.0263078.s008.docx]

| **Reviewer's Comment** | **Response** |
| --- | --- |
| **Background** | |
| 1. What does it mean “retrospective approach” in line 83-84. | As the data was collected based on past history of their COVOD-19 infection and vaccination as we mentioned in the method. |
| 2. Need to mention more detail about the student participants to get clear presentation for readers. Not enough described in line 95-96 and 100-101. | Already Mentioned in line 95-96 and 100-101 |
| 3. What is the total number of students in in summer-2021 semester (May to August 2021) of the MPH program of NUB? Are public health students being part-time students? Are they also working? | Modified in line 91-95 |
| 4. The sample size said 384 but the study enrolled 300 students? What is your explanation for that? | Already Mentioned in line 101-103 |
| 5. In data analysis, AOR should be used instead of OR in multivariable logistic regression. | Modified in line 133 |
| **Editor's Comment** |  |
| Minor grammatical errors throughout the manuscript require correction. | Already addressed |
